# Pharmacokinetics of SPR206 in Plasma, Pulmonary Epithelial Lining Fluid, and Alveolar Macrophages following Intravenous Administration to Healthy Adult Subjects

**DOI:** 10.1128/aac.00426-23

**Published:** 2023-06-20

**Authors:** Keith A. Rodvold, Justin Bader, Jon B. Bruss, Kamal Hamed

**Affiliations:** a University of Illinois Chicago, Chicago, Illinois, USA; b Spero Therapeutics, Inc., Cambridge, Massachusetts, USA

**Keywords:** SPR206, alveolar macrophage, epithelial lining fluid, pharmacokinetics, pulmonary

## Abstract

SPR206 is a next-generation polymyxin being developed for the treatment of multidrug-resistant (MDR) Gram-negative infections. This Phase 1 bronchoalveolar lavage (BAL) study was conducted to evaluate SPR206’s safety and pharmacokinetics in plasma, pulmonary epithelial lining fluid (ELF), and alveolar macrophages (AM) in healthy volunteers. Subjects received a 100 mg intravenous (IV) dose of SPR206 infused over 1 h every 8 h for 3 consecutive doses. Each subject underwent 1 bronchoscopy with BAL at 2, 3, 4, 6, or 8 h after the start of the third IV infusion. SPR206 concentrations in plasma, BAL, and cell pellet were measured with a validated LC-MS/MS assay. Thirty-four subjects completed the study and 30 completed bronchoscopies. Mean SPR206 peak concentrations (*Cmax*) in plasma, ELF, and AM were 4395.0, 735.5, and 860.6 ng/mL, respectively. Mean area under the concentration-time curve (AUC_0-8_) for SPR206 in plasma, ELF, and AM was 20120.7, 4859.8, and 6026.4 ng*h/mL, respectively. The mean ELF to unbound plasma concentration ratio was 0.264, and mean AM to unbound plasma concentration ratio was 0.328. Mean SPR206 concentrations in ELF achieved lung exposures above the MIC for target Gram-negative pathogens for the entire 8-h dosing interval. Overall, SPR206 was well tolerated; 22 subjects (64.7%) reported at least 1 treatment-emergent adverse event (TEAE). Of the 40 reported TEAEs, 34 (85.0%) were reported as mild in severity. The most frequent TEAEs were oral paresthesia (10 subjects [29.4%]) and nausea (2 subjects [5.9%]). This study demonstrates pulmonary penetration of SPR206 and supports further development of SPR206 for the treatment of patients with serious infections caused by MDR Gram-negative pathogens.

## INTRODUCTION

Antimicrobial resistance is a growing problem worldwide, and carbapenem-resistant Acinetobacter baumannii, multidrug-resistant (MDR) Pseudomonas aeruginosa, and carbapenem-resistant and extended-spectrum beta-lactamase (ESBL)-producing *Enterobacterales* have been identified as urgent or serious threats by the CDC and WHO (1, 2). In particular, bacteremia, hospital-acquired and ventilator-associated bacterial pneumonia (HABP/VABP), and complicated urinary tract infections are often caused by A. baumannii (3), frequently MDR strains (4–7), and these infections account for excess morbidity and mortality (3, 8–15). Similarly, P. aeruginosa causes serious systemic infections with increased rates of morbidity and mortality (16, 17).

The continuing emergence and spread of MDR ESBL- and carbapenemase-producing clinical isolates of *Enterobacterales*, P. aeruginosa, and A. baumannii are limiting available options to treat infections caused by these pathogens. At present, most carbapenemase-producing strains of Klebsiella pneumoniae, Escherichia coli, P. aeruginosa, and A. baumannii remain susceptible to polymyxin antibiotics (polymyxin B and colistin), and a resurgence is seen in the use of these drugs for the treatment of serious Gram-negative infections. However, toxicity, particularly nephrotoxicity and neurotoxicity, restricts the use of polymyxins (18, 19). These findings highlight the urgent need to identify new antimicrobial agents to treat serious infections due to MDR Gram-negative pathogens (1, 2, 20–24).

SPR206 is a polymyxin derivative with activity against many Gram-negative pathogens, including A. baumannii, P. aeruginosa, K. pneumoniae, E. coli, and Enterobacter spp. that pose an increased risk for antimicrobial resistance (25–30). *In vitro* and *in vivo* studies suggest that the activity of SPR206 is more potent compared to polymyxin B (31–33). Additionally, nonclinical studies suggest the potential for an improved safety profile with SPR206 compared to polymyxin B (25, 34, 35). SPR206 was generally safe and well tolerated at exposures required for efficacy, with a low risk for respiratory, central nervous system, or cardiovascular events and a low risk for clinically significant drug-drug interactions (34, 35). SPR206 is being developed for intravenous (IV) administration for the treatment of serious infections of the respiratory tract, namely, HABP/VABP due to drug-resistant Gram-negative pathogens. A first-in-human pharmacokinetic (PK) and safety study demonstrated no appreciable drug accumulation with repeated 100 mg IV doses q8h of SPR206 for 14 days and no evidence of nephrotoxicity (36).

Concentrations of antibiotics in pulmonary epithelial lining fluid (ELF) and in alveolar macrophages (AM) are important for determining antibiotic activity and optimal dosing in patients with pneumonia (37, 38). This study was designed to determine the concentrations of SPR206 in ELF and AM compartments of the lung to provide essential information for the development of SPR206 as an antibacterial agent for the treatment of lower respiratory tract infections. The primary objectives of this study were to evaluate the safety and the intrapulmonary PK, including ELF and AM concentrations, of SPR206 compared to plasma concentrations of SPR206 in healthy adult subjects.

## RESULTS

Thirty-four subjects were enrolled in the study, and all were dosed and included in the safety and PK populations. Seven subjects were excluded from the BAL PK analysis for protocol deviations (3) or lack of completed BAL samples (4), leaving 27 subjects in the BAL PK analysis. Baseline characteristics are shown in Table 1.

**TABLE 1:** Baseline characteristics (safety population)

Characteristic	Subjects (N = 34)
Age, yrs^*a*^	41.9 ± 8.7
Age range, yrs	24–55
Male, n (%)	24 (70.6)
Race, n (%)	
Asian	3 (8.8)
White	30 (88.2)
Not reported	1 (2.9)
Not Hispanic or Latino, n (%)	32 (94.1)
wt, kg^*a*^	79.4 ± 12.4
Body mass index, kg/m^2^^*a*^	26.4 ± 2.8

a Mean ± standard deviation.

### Pharmacokinetics.

**FIG 1:**
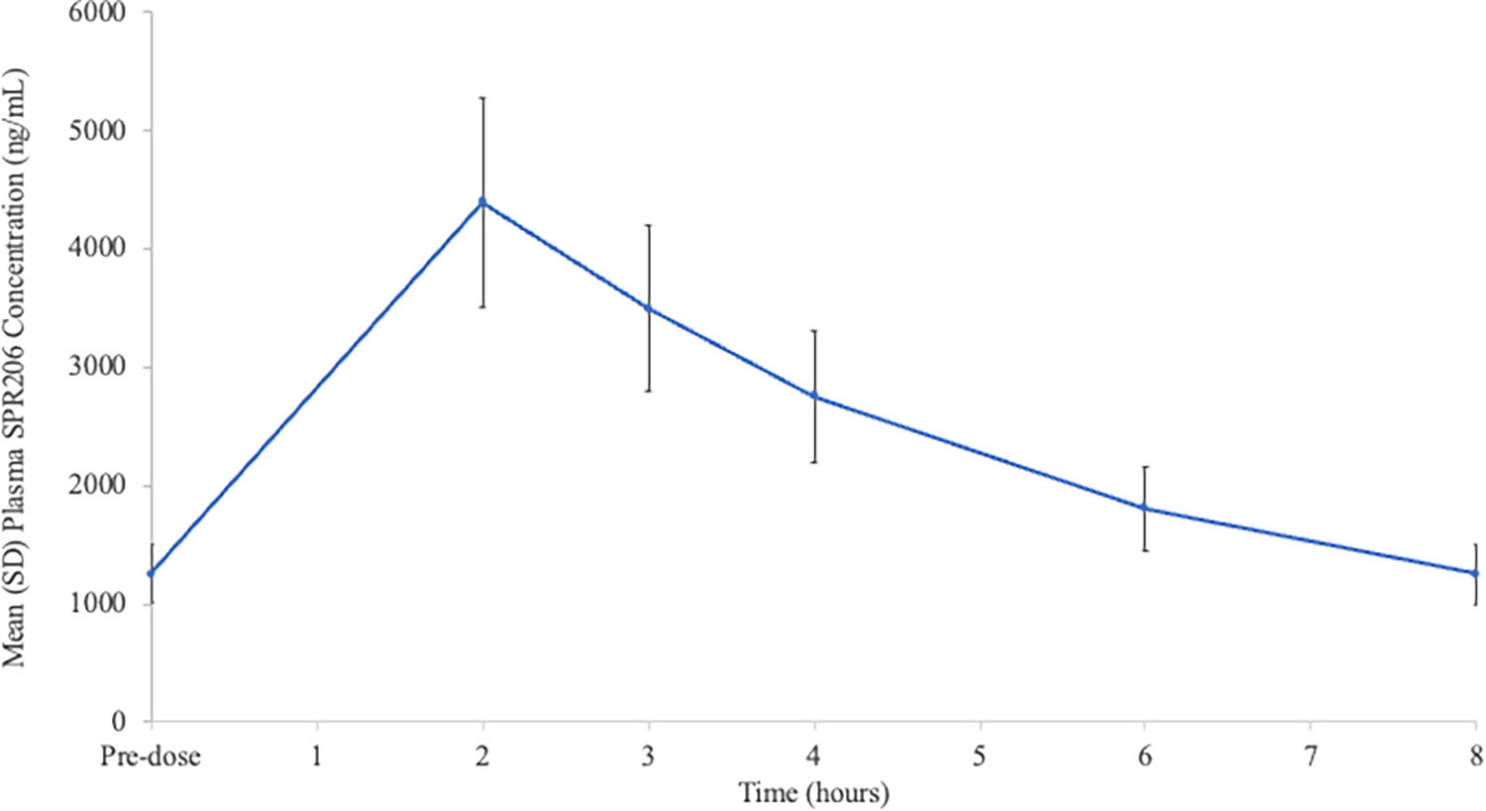
Mean (SD) plasma (total) SPR206 concentrations after 100 mg intravenously every 8 h for 3 doses.

Following administration of SPR206 100 mg IV q8h, plasma SPR206 concentrations peaked at 2 h following the third dose, and thereafter declined reaching pre-dose concentrations at 8 h after the third dose (Fig. 1). The similarity of SPR206 concentrations prior to the third dose and at 8 h following that dose indicated that steady-state had been achieved. Median time to peak plasma concentrations (*T*_max_) occurred at 2 h (range: 1.8 h to 2.9 h) (Table 2).

**TABLE 2:** Plasma pharmacokinetic (PK) parameters obtained after the third SPR206 100 mg intravenous dose (PK population)^*a*^

Parameter	Arithmetic mean (SD^*b*^)	Geometric mean (CV^*c*^%)
AUC_0-8_ (h*ng/mL)^*d*^	20120.7 (3555.2)	19817.9 (17.9)
*C*_max_^*e*^ (ng/mL)	4395.0 (752.7)	4333.4 (17.2)
*C*_min_^*f*^ (ng/mL)	1409.9 (709.4)	1306.9 (36.7)
*T*_max_^*g*^ (hours)	2.1 (0.2)	2.1 (7.4)
*t*_1/2_^*h*^ (hours)	3.6 (0.5)	3.6 (13.5)
*V*_z_^*i*^ (mL)	19806.1 (3533.7)	19502.1 (18.1)

a PK population included all the subjects who received at least one dose of SPR206 and had at least one evaluable plasma concentration. d, h, and i, N = 32; e, f, and g, N = 34.

b SD, standard deviation.

c CV, coefficient of variation.

d AUC_0-8_, area under the concentration-time curve from time zero to 8 h.

e *C*_max_, maximum plasma concentration.

f *C*_min_, minimum plasma concentration.

g *T*_max_, time to *C*_max_.

h *t*_1/2_, half-life.

i *V*_z_, volume of distribution at the terminal phase.

**FIG 2:**
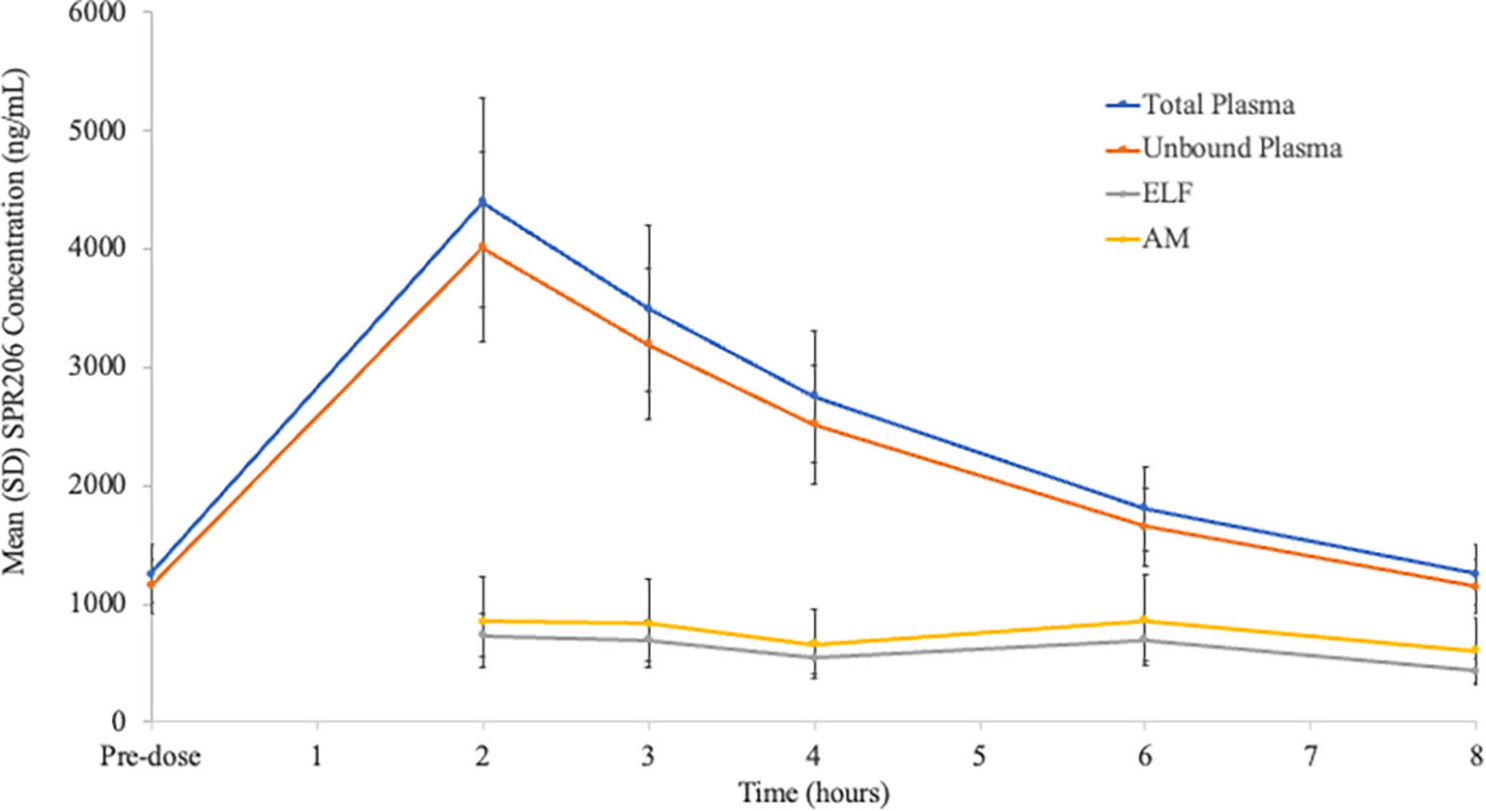
Mean (SD) SPR206 concentrations in plasma (total and unbound), ELF, and AM at BAL sampling time points after the third 100 mg intravenous dose. AM, alveolar macrophages; ELF, epithelial lining fluid.

In the BAL PK Population, 3 doses of SPR206 100 mg IV q8h produced steady-state concentrations in ELF and AM (Fig. 2) with concentration-time profiles of SPR206 for ELF and AM that were nearly flat. At each post-dose BAL sampling time point, the mean SPR206 concentration in AM (range: 604.2 ng/mL to 860.6 ng/mL) was slightly higher than the ELF concentration (range: 431.5 ng/mL to 735.5 ng/mL) (Table 3). Mean area under the concentration-time curve from time zero to 8 h (AUC_0-8_) for SPR206 in ELF and AM was 4859.8 h*ng/mL and 6026.4 h*ng/mL, respectively. Mean peak concentrations (*Cmax*) for SPR206 in ELF and AM were 735.5 ng/mL and 860.6 ng/mL, respectively, and mean minimum concentration (*C*_min_) in ELF and AM was 431.5 ng/mL and 604.2 ng/mL, respectively. The *T*_max_ in ELF was 2.2 h; however, the *T*_max_ was delayed to 6.4 h in AM.

**TABLE 3:** Ratio of ELF and AM concentrations to total and unbound plasma concentrations of SPR206^*a*^

	Arithmetic mean ± SD			
BAL^*b*^ sampling time post-dose (hours)	ELF^*c*^ to total plasma	ELF to unbound plasma	AM^*d*^ to total plasma	AM to unbound plasma
2	0.183 ± 0.032	0.200 ± 0.035	0.206 ± 0.114	0.226 ± 0.124
3	0.199 ± 0.066	0.218 ± 0.072	0.184 ± 0.065	0.201 ± 0.071
4	0.190 ± 0.067	0.208 ± 0.074	0.223 ± 0.089	0.244 ± 0.097
6	0.428 ± 0.176	0.469 ± 0.192	0.519 ± 0.223	0.568 ± 0.244
8	0.347 ± 0.138	0.380 ± 0.151	0.503 ± 0.192	0.550 ± 0.210

a PK Population included all the subjects who received at least one dose of SPR206 and had at least one evaluable plasma concentration. The BAL PK Population included all the subjects who received at least one dose of SPR206 and had at least one evaluable BAL concentration. Ratios were calculated by dividing individual SPR206 concentration in ELF or AM by corresponding total and unbound plasma SPR206 concentration.

b BAL, bronchoalveolar lavage.

c ELF, epithelial lining fluid.

d AM, alveolar macrophages.

Both ELF and AM concentrations were lower than total and unbound SPR206 plasma concentrations over the 8-h sampling period after the last dose (Fig. 2). SPR206 penetration ratios in ELF and AM relative to unbound plasma concentrations ranged from 0.200 to 0.469 for ELF and 0.201 to 0.568 for AM (Table 3). Furthermore, the ratios of SPR206 in AM to unbound plasma concentrations were slightly higher compared with ratios of ELF to unbound plasma concentrations for most BAL sampling time points (Table 4).

**TABLE 4:** Ratios of ELF and AM AUC_0-8_ to total and unbound plasma AUC_0-8_ following SPR206^*a*^

Ratio of AUC_0-8_^*b*^ of ELF^*c*^ to AUC_0-8_ of total plasma	Ratio of AUC_0-8_ of ELF to AUC_0-8_ of unbound plasma	Ratio of AUC_0-8_ of AM^*d*^ to AUC_0-8_ of total plasma	Ratio of AUC_0-8_ of AM to AUC_0-8_ of unbound plasma
0.242	0.264	0.300	0.328

a PK Population included all the subjects who received at least one dose of SPR206 and had at least one evaluable plasma concentration. The BAL*^e^* PK Population included all the subjects who received at least one dose of SPR206 and had at least one evaluable BAL concentration. The AUC_0-8_ of total and unbound plasma was calculated using PK Population (*n* = 34). The AUC_0-8_ of ELF and AM was calculated using BAL PK Population (*n* = 27).

b AUC_0-8_, area under the concentration-time curve from time zero to 8 h.

c ELF, epithelial lining fluid.

d AM, alveolar macrophages.

e BAL, bronchoalveolar lavage.

### Safety.

Treatment with SPR206 was safe and well tolerated in healthy subjects. Overall, 64.7% of subjects experienced at least 1 treatment-emergent adverse event (TEAE) and 13 subjects (38.2%) experienced at least 1 treatment-related TEAE. Of the 40 reported TEAEs, 34 (85.0%) were reported as mild in severity. The most frequent treatment-related TEAEs were oral paresthesia in 10 (29.4%) subjects and nausea in 2 (5.9%) subjects. All paresthesia-like events (paresthesia or hypoesthesia) were mild. No deaths, serious AEs, or AEs leading to study drug discontinuation or study discontinuation were reported. No clinically significant abnormalities were reported for clinical laboratory parameters including blood urea nitrogen, serum creatinine, and estimated creatinine clearance. No clinically significant changes were noted for vital signs, electrocardiogram (ECG) assessments, or physical examinations.

## DISCUSSION

The PK analysis of SPR206 plasma concentrations in this study are consistent with previous findings from a Phase 1 ascending dose study of SPR206 in healthy subjects (36). In the single SPR206 100 mg IV dose cohort, *Cmax* was 5300 ng/mL and AUC_0-inf_ was 20400 h*ng/mL, which was comparable to the *Cmax* of 4300 ng/mL and steady-state AUC_0-8_ of 19800 h*ng/mL reported here. The lower values of *Cmax* and AUC_0-8_ observed in this study were likely explained by differences in the number blood samples (13 versus 6) and the absence of a sampling time at the end of the 1-h infusion. The data from this study will be incorporated into a population PK model under development and assessed relative to pharmacokinetic/pharmacodynamic targets for efficacy to provide additional support for probability of PK target attainment and dose selection for future studies.

The results of this study provided important information on intrapulmonary PK of SPR206 in healthy subjects. Estimated intrapulmonary SPR206 penetration ratios determined from the ratio of AUC_0-8_ in ELF and AM to unbound plasma SPR206 concentrations were 0.264 and 0.328, respectively. Concentrations of SPR206 in ELF (range: 431.5 ng/mL to 735.5 ng/mL) and AM (range: 604.2 ng/mL to 860.6 ng/mL) were higher than the observed MIC_90_ of SPR206 against most contemporary Gram-negative organisms (≤ 500 ng/mL) based on data from *in vitro* and *in vivo* studies (26, 27, 30, 33, 39, 40). While ELF and AM concentrations were determined in healthy subjects in this study, intrapulmonary concentrations are expected to be higher in patients with respiratory infections, such as pneumonia (41). Administration of 3 doses of SPR206 100 mg IV q8h was well tolerated. Similar to the previous study, mostly mild paresthesias were the most common AE with no deaths or serious AEs and no clinically significant abnormalities observed for laboratory parameters, vital signs, ECG assessments, or physical examination.

Intrapulmonary concentrations of systemically administered antibiotics that exceed the MIC of target pathogens are important to achieve successful treatment outcomes from serious infections of the respiratory tract, especially those patients with pneumonia and in critical care units (37, 41–43). Beta-lactams, fluoroquinolones, macrolides, linezolid, and tigecycline demonstrate good penetration following oral or parenteral administration (41, 43, 44). In contrast, lung penetration with colistin and polymyxin B following IV administration is poor and highly variable. In animal models, lung concentrations of colistin were undetectable (45) or below the MIC_50_ for P. aeruginosa (46). Binding of colistin to mucin in airways results in subtherapeutic concentrations in the lung even with IV administration and could potentially promote development of resistance (47, 48). While data are limited for polymyxin B and colistin, IV administration of colistin in critically ill patients resulted in undetectable levels in BAL fluid (49), which may have been the result of extensive tissue binding (50). Additionally, achieving an adequate dose of polymyxin B or colistin to reach a bactericidal concentration in the target tissue can pose a challenge because of the potential for nephrotoxicity (46, 48, 51).

Serious infections due to MDR Gram-negative bacteria often are treated with aminoglycosides, carbapenems, and polymyxins. However, use of aminoglycosides and polymyxins is limited by serious side effects. Aminoglycosides and polymyxins are associated with an increased risk for nephrotoxicity (19, 52, 53). In addition, aminoglycosides increase the risk of ototoxicity, and polymyxins are associated with an increased risk for neurotoxicity (54). Both aminoglycosides and polymyxins require therapeutic drug monitoring to confirm that optimal drug concentrations are achieved and to avoid serious toxicity (55). Even at recommended dosages of polymyxins, acute kidney injury may occur in up to 60% of patients (56), and all-cause nephrotoxicity was reported in over 40% of patients and renal failure in 11.2% (18).

SPR206 is a novel polymyxin B analogue, which was developed with the goal of limiting the nephrotoxicity potential of polymyxins (34) while maintaining the *in vitro* and *in vivo* activity against MDR Gram-negative pathogens. Results from nonclinical studies showed that SPR206 reduced kidney cell cytotoxicity and had lower exposure in the kidney than polymyxin B (34, 35). These results together with results from Phase 1 studies (36, 57) suggest the potential of SPR206 to offer a broad spectrum of activity against MDR pathogens, but with an improvement in the safety profile compared with older polymyxins.

In summary, this study demonstrated that IV administration of SPR206 achieves plasma concentrations that were consistent with previous studies in healthy volunteers and exhibits lung penetration into ELF and AM. Concentrations of SPR206 in ELF and AM exceeded the MIC_90_ for most Gram-negative pathogens causing serious bacterial infections including A. baumannii, K. pneumoniae, and P. aeruginosa. These results support evaluation of SPR206 for treating serious respiratory infections caused by MDR Gram-negative pathogens.

## MATERIALS AND METHODS

The study was conducted between May 2021 and September 2021 at the Medicines Evaluation Unit, Ltd., Manchester, United Kingdom in accordance with the U.S. Code of Federal Regulations and ethical principles of the Declaration of Helsinki, Good Clinical Practices, and the International Council for Harmonisation guidelines. The study protocol and all amendments were reviewed by the Institutional Review Board for the 2 study centers (NHS Health Research Authority, South Central - Berkshire Research Ethics Committee, Bristol, United Kingdom). Informed consent was obtained from each subject in writing before any study procedures were performed. This study was registered at clinicaltrials.gov: NCT04868292.

### Study design.

This was a Phase 1, single-center, multiple-dose, open-label PK study in healthy adult male and female subjects. Subjects received three 100 mg doses of SPR206 administered q8h as a 1-h IV infusion. Three consecutive doses were sufficient to achieve steady-state based on a previous Phase 1 study in healthy subjects (36). A screening evaluation to determine eligibility for enrollment into the study was performed within 28 days of initial dosing. Subjects who met inclusion and exclusion criteria returned to the study unit 1 day before dosing (Day -1). Subjects who met inclusion and exclusion criteria were confined to the study site from Day -1 (1 day prior to start of dosing) and remained in the study site through completion of all scheduled procedures including collection of the last PK sample and bronchoscopy procedures along with safety evaluations (Day 2). Each subject underwent 1 bronchoscopy with BAL at 2, 3, 4, 6, or 8 h after the start of the third IV infusion. Subjects were required to return for a follow-up visit on Day 7 (+2 days) after discharge from the study site. The maximum duration of participation for each subject was up to 39 days (up to 28 days for Screening, 2 days of confinement, and up to 9 days for follow-up).

### Study population.

Eligible subjects were adults aged 18 to 55 years with a body mass index (BMI) ≥18.5 and ≤32 kg/m^2^ and body weight between 55.0 and 100.0 kg who had been nonsmokers for at least 12 months prior to screening. Subjects were medically healthy without clinically significant abnormalities based on a screening medical history, physical examination, vital signs, 12-lead ECG, and clinical laboratory tests. Subjects had a forced expiratory volume in 1 s (FEV_1_) of at least 80% of predicted at screening; abstained from alcohol, caffeine, xanthine-containing beverages, or food for 48 h prior to and during the study; and agreed to use an effective method of contraception during the study.

At screening, subjects were excluded for a history of any significant medical condition; history (within 6 months) of known or suspected Clostridioides difficile infection; positive urine drug, alcohol, or cotinine testing; positive test for human immunodeficiency virus (HIV), hepatitis B surface antigen (HBsAg), or hepatitis C antibodies (HCV Ab); positive test for severe acute respiratory syndrome coronavirus 2 (SARS-CoV-2); presence of fever, chills, or sweats; difficulty breathing; cough; sore throat; loss of taste or smell; or nausea, vomiting, or diarrhea or within 28 days prior to screening. Subjects with an ECG finding of QTcF interval duration ≥450 msec for males and 470 msec for females were excluded as were those with any clinical laboratory abnormalities.

### Blood sample collection.

Blood samples for determining SPR206 plasma concentrations were collected within 60 min pre-dose (0 h) prior to second and third doses of SPR206 only and at 2, 3, 4, 6, and 8 h after the start of the third dose of SPR206.

### Bronchoscopy and BAL.

Subjects were assigned to 1 of 5 bronchoscopy sampling times at 2, 3, 4, 6, or 8 (± 10 min) after the third IV infusion of SPR206. Each subject underwent 1 standardized bronchoscopy and BAL on Day 2 at the assigned time. A blood sample to determine plasma concentrations of SPR206 and urea was obtained during the bronchoscopy procedure at each BAL sampling time (approximating the time of collection of a second aspirate of BAL). Detail descriptions of the outpatient bronchoscopy, BAL procedures, and the handling, processing, and storage of samples have been previously described (58–60).

### Determination of plasma concentrations of SPR206.

Concentrations of SPR206 in plasma were determined by a validated liquid chromatography with tandem mass spectrometry (LC-MS/MS) assay performed at QPS, LLC. For plasma, the assay range was 50 to 50,000 ng/mL. In plasma, intraday precision (% coefficient of variation) ranged from 1.3% to 7.7%, and accuracy (% relative error) ranged from -1.2% to 9.5%. Interday precision ranged from 2.9% to 5.3% and accuracy ranged from 1.6% to 6.0%.

### Determination of BAL fluid and cell pellet concentrations of SPR206.

The concentrations of SPR206 in BAL and in cell pellets were determined by Keystone Bioanalytical, Inc. using a validated LC-MS/MS method. ELF concentrations were calculated by the urea dilution method (61, 62). AM concentrations were determined from cell pellet drug concentrations, cell count in BAL fluid, and macrophage cell volume (62). The calibration curves for SPR206 were linear over a range from 2 to 1,000 ng/mL. The inter-assay precision (%CV) and accuracy (%RE) for SPR206 were 2.81% to 15.84% and -4.02% to 1.51%, respectively. The intra-assay precision (%CV) and accuracy (%RE) for SPR206 were 1.43% to 18.59% and -17.44% to 6.28%, respectively.

### Determination of urea concentration.

The concentration of urea in plasma and BAL fluid supernatants was determined using a validated LC/MS/MS method from Keystone Bioanalytical, Inc. Thirty human plasma and 30 BAL fluid samples were assayed for urea concentrations. A total of 10 samples for both plasma and BAL fluid (33.3%) were selected for testing, and the calculated assay variability was within 20%. The calibration curve for the urea assay in plasma was linear over the range from 100 to 3,000 μg/mL. The inter-assay precision (%CV) and accuracy (%RE) for SPR206 were 1.98% to 3.37% and -8.17% to 4.13%, respectively. The intra-assay precision (%CV) and accuracy (%RE) for SPR206 were 0.56% to 5.60% and -10.21% to 8.54%, respectively. The calibration range of the urea assay for BAL fluid was linear (*r*^2^ > 0.998) over the range of 0.2 to 10 μg/mL. The inter-assay precision (%CV) and accuracy (%RE) for SPR206 were 6.40% to 9.77% and -3.22% to 1.47%, respectively. The intra-assay precision (%CV) and accuracy (%RE) for SPR206 were 0.96% to 12.08% and -11.30% to 9.15%, respectively.

### Pharmacokinetic analysis.

PK analysis of SPR206 plasma concentrations was determined with noncompartmental PK analysis using Phoenix WinNonlin software (version 8.3, Certara Inc.). PK parameters were *T*_max_, *Cmax*, *C*_min_, and AUC_0-8_.

### ELF volume and antibiotic concentrations for ELF and AM.

Actual BAL samples were collected outside the protocol-specified sampling time windows. Thus, an updated method was utilized where the PK parameters for ELF or AM were calculated from composite concentration profiles of ELF or AM, respectively, based on the 27 subjects in the BAL PK population. Some blood samples were collected outside of the planned sampling windows, and therefore, individual plasma AUC_0-8_ was calculated first and the mean population plasma AUC_0-8_ was derived from all individual AUC_0-8_ values.

Concentrations of SPR206 were summarized separately for plasma, ELF, and AM by nominal time point using descriptive statistics. The mean concentrations of SPR206 in BAL fluid obtained at the different BAL fluid sampling times (e.g., 2, 3, 4, 6, and 8 h) were used to estimate the AUC_0-8_ for SPR206 in plasma, ELF, and AM. The concentration at the final sampling time (8 h) also served as the time zero value for determining the AUC_0-8_ value of each matrix using Phoenix WinNonlin software (version 8.3, Certara Inc.). The ratios of the AUC_0-8_ of ELF or AM to the AUC_0-8_ of plasma (total and unbound) were calculated. Plasma protein binding analyses were not conducted as part of this study. The value used for the unbound fraction of SPR206 in plasma was 0.914 (Spero Therapeutics, Inc., data on file). The measured concentrations in ELF and AM represented unbound concentrations, since only unbound plasma fractions are considered to penetrate the lung compartments. Individual plasma concentration-time profile versus time and arithmetic mean concentration (±SD) versus time plots were presented for the concentration-time data.

Calculations of the ELF volume and drug concentrations in ELF and AM were performed with BAL fluid supernatants and cell pellets from pooled aspirates (60). The concentration of SPR206 in ELF (C_ELF_) was determined as follows: C_ELF_ = concentration in BAL × BAL volume/ELF volume. ELF volume was derived from ELF volume = BAL volume × urea_BAL_/urea_plasma_, where urea_BAL_ was the concentration of urea in BAL fluid and urea_plasma_ was the concentration of urea in plasma.

The concentration of SPR206 in AM (C_AM_) was determined using the equation C_AM_ = C_pellet_ suspension/V_AM_, where C_pellet_ is the mass of SPR206 measured in the cell suspension and V_AM_ is the volume of alveolar cells in the 1-mL cell suspension. A mean macrophage cell volume of 2.42 μL/10^6^ cells was used for V_AM_ (63, 64).

### Statistical analysis.

The study was primarily a descriptive study comparing the steady-state concentrations of SPR206 in plasma, ELF, and AM at selected time intervals. Thus, no formal statistical hypothesis testing was performed. The sample size was based on the need to obtain adequate safety, tolerability, and PK data to achieve the objectives of the study while exposing as few subjects as possible to study medication and procedures. The sample size of 6 subjects per cohort was considered sufficient to provide adequate PK data at each BAL time point. SAS Version 9.4 was used to analyze the data, and create summary tables, subject data listings, and graphical representations of data.

### Safety assessments.

Subjects were continuously monitored during the bronchoscopy. Vital signs (blood pressure, heart rate, and respiratory rate) were recorded prior to the scheduled bronchoscopy time and at 15 min and 1 h following the end of bronchoscopy. Subjects were kept under observation for 2 to 3 h post-bronchoscopy for safety evaluations. Continuous pulse oximetry was conducted from the beginning of preparation of the subject for bronchoscopy until the post-procedure oxygen saturation was at least 93% on room air. Pulse oximetry was extended if a clinically significant increase in heart rate or cardiac rhythm abnormalities were detected. Other safety assessments included physical examinations, clinical laboratory monitoring (hematology, blood chemistry, and urinalysis), 12-lead ECG, and adverse event recording.
